# Stimulus-Dependent Inhibitor of Apoptosis Protein Expression Prolongs the Duration of B Cell Signalling

**DOI:** 10.1038/srep27706

**Published:** 2016-06-09

**Authors:** Hisaaki Shinohara, Kentaro Inoue, Noriko Yumoto, Takeshi Nagashima, Mariko Okada-Hatakeyama

**Affiliations:** 1 Laboratory for Integrated Cellular Systems, RIKEN Center for Integrative Medical Sciences (IMS), Yokohama, Kanagawa 230-0045, Japan

## Abstract

Different dynamic behaviours of signalling activity can induce distinct biological responses in a variety of cells. However, the molecular mechanisms that determine the dynamics of kinase activities in immune cells are not well understood. In this study, we showed that the duration of both IκB kinase (IKK) and extracellular signal-regulated kinase (ERK) activities in B cell receptor (BCR)- and CD40-signalling pathways in B cells were regulated by transcriptional feedback loops. We conducted a time-course transcriptome analysis after BCR or CD40 stimulation and identified the following four candidate genes as feedback regulators for IKK and ERK: inhibitor of apoptosis protein (*IAP*), TNF alpha-induced protein 3, dual-specificity phosphatase 5, and sprouty homolog 2. Quantitative experiments and mathematical modelling suggested that IAP inhibition shortened the duration of IKK and ERK activity following both BCR and CD40 pathway stimulation, indicating a positive role for IAP in B cell signalling. Furthermore, transient kinase activities induced by IAP blockage reduced the levels of delayed expression genes. Together, our findings suggest that IKK and ERK activity durations can be fine-tuned by the coordinated regulation of positive and negative transcriptional feedback and that these network properties determine the biological output of B cells.

The duration of kinase activity is crucial in deciding the fate of mammalian cells. It is widely known that the transient and sustained activation of extracellular signal-regulated kinase (ERK) induces the proliferation and differentiation of many types of cells^1^,^2^,^3^,^4^,^5^,^6^ and that prolonged IκB kinase (IKK) activity is required for stimulus-specific gene expression programs^7^. Furthermore, in the immune system, sustained signalling activities are important for efficient T cell activation and differentiation^8^,^9^,^10^,^11^,^12^,^13^,^14^,^15^. Although the kinetics of different molecules are involved in determining the duration of signalling activities^16^,^17^, feedback regulation in the signalling network also plays a role in duration control. Two types of feedback regulation are known to determine the duration of signalling activities: rapid post-translational feedback (mainly caused by protein phosphorylation or dephosphorylation) and slow transcriptionally induced feedback. The ERK system is a typical example wherein activity is dynamically controlled by these two types of feedback loops through post-translational modifications^4^,^5^ and transcription-induced regulation^6^,^18^. Transcriptionally induced feedback regulators modulate signalling activity on a longer timescale; therefore, their functions in the control of cell fate appear to be of particular importance^18^.

B cells play an important role in humoral adaptive immunity. The recognition of foreign antigens by B cell receptors (BCRs) initiates multiple signalling cascades for the induction of B cell activation, expansion, differentiation, and cell survival, which result in mobilisation aimed at antigen clearance^19^. Importantly, dysregulation of these processes leads to immunodeficiency, lymphoid tumours, or autoimmune disease^20^. After the engagement of a BCR with an antigen, secondary signals mediated by co-stimulatory molecules such as CD40, a tumour necrosis factor (TNF) receptor family member, promote adequate development of the adaptive immune response. The association of CD40 with its ligand, CD40L/CD154 (which is transiently expressed on T cells) functions to permit and promote B cell survival, proliferation, and development^21^. Thus, the general activation scheme of B cell signalling has been well defined; however, the activation dynamics and mechanisms which control individual signalling pathways that respond to BCR- and CD40-stimulation have not yet been explicitly clarified.

The BCR and CD40 signalling pathways share IKK-nuclear factor (NF)κB^22^ and ERK core modules. These two signalling activities are crucial for B cell activation processes^23^,^24^ and are controlled by feedback regulation. IKK-NF-κB, A20 (TNF alpha-induced protein 3 (*TNFAIP3*)), and IκBs have been reported to serve as general negative feedback regulators that can attenuate pathway signalling^7^,^25^. Dual-specificity phosphatase (DUSP) is known to have a similar function in controlling ERK^24^,^25^. Notably, these genes are expressed very early in the response to cell stimulation, thereby enabling control of the dynamics of upstream signalling pathways. This suggests that other genes that are immediately expressed in response to receptor activation might also have as-yet unknown functions toward regulating the dynamics of BCR- and CD40-signalling.

Understanding the mechanisms that control signalling pathways is important for decisions related to cell fate and the immune response; however, the identification of mechanisms involved in feedback regulation is difficult to achieve solely by gene-targeting experiments. Because signalling activity is controlled by multi-layered positive and negative feedback loops associated with different time constants, computer simulations combined with quantitative experiments represent a more relevant approach to uncover mechanisms involved in the control of signalling networks^26^. To assess the signalling dynamics of kinases, as well as the regulatory functions of transcriptional feedback, we investigated the time-course of gene expression in B cells up to 90-min after BCR- or CD40-stimulation. As a result, we identified the early response gene, baculoviral IAP repeat-containing 2 (*BIRC2*), also called cellular inhibitor of apoptosis 1 (*cIAP1)*, hereinafter called *IAP*, as a positive feedback regulator of ERK and IKK. We showed that the dynamics of ERK and IKK activities in response to BCR- and CD40- stimulation were distinct, but that they could both be positively modulated by the induction of IAP. Furthermore, mathematical modelling combined with experimentation demonstrated that the sustained activities of ERK and IKK were required for late-phase *Bcl2l1* and *Pik3r* gene expression. Our studies suggest that the dynamic cross-talk between signalling and transcription processes is important to determine the B cell gene expression output that is involved in the immune response.

## Results

### Dynamics of BCR- and CD40-induced IKK and ERK activation in B cells

**Figure 1:**
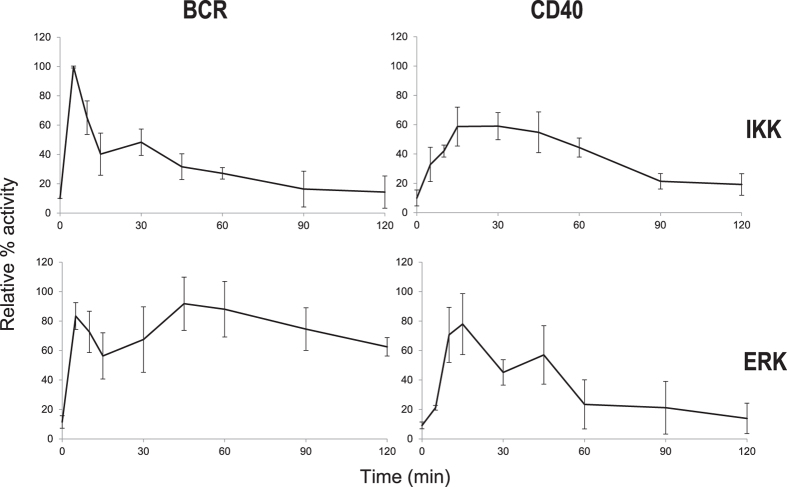
Activation dynamics of IKK or ERK induced by BCR or CD40. Quantification of IKK and ERK activity induced by anti-IgM (BCR) or anti-CD40 (CD40) as analysed by immuno blotting in Supplementary Fig. S1; data represent the means ± s.d. (n = 2).

We examined the dynamics of ERK and IKK activities over time (up to 120 min) following BCR or CD40 stimulation with anti-IgM or CD40 ligands, respectively (Fig. 1, Supplementary Fig. S1). Following BCR application, B cells from a mouse spleen (Splenic B) showed rapidly decaying IKK activation and sustained activation of ERK. On the other hand, CD40 stimulation induced sustained IKK and transient ERK activities (Fig. 1). Among these findings, we observed a characteristically prolonged duration of BCR-stimulated ERK activity at later time points. Similar activation patterns of IKK and ERK were observed in the DT40 B cell line (Supplementary Fig. S1), which has been shown to be suitable for the comprehensive analysis of regulatory networks^27^.

### Gene expression profiles of BCR and CD40-stimulated B cells

**Figure 2:**
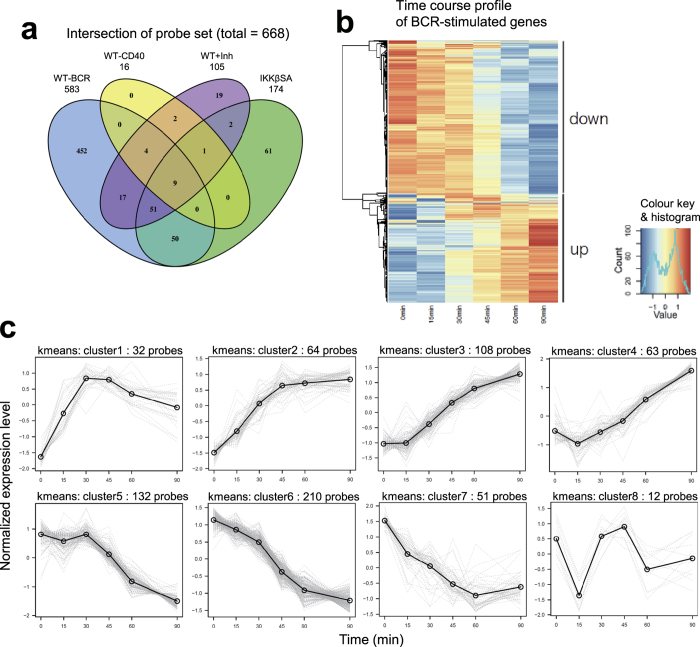
Gene expression analysis by microarray. (**a**) DT40 B cells were stimulated with anti-IgM or CD40L and subjected to microarray expression analysis. Venn diagram illustrating the gene expression profiles of wild type cells from anti-IgM treated (WT-BCR) or CD40L ligation (WT-CD40) and of MEK inhibitor treated (WT-Inh) or IKKβ inactive cells (IKKβSA). (**b**) Time course clustering of gene expression data. Wild type cells were stimulated with 10 μg/ml anti-IgM (M4) for the indicated time periods. The relative gene expression is depicted as a heat map according to the colour scale shown. The count represents the number of genes with expression levels presented in the X-axis. The horizontal axis of the value represents the normalized expression number of the genes. (**c**) k-means clustering. The time course profiles in Fig. 2b were subjected to k-means clustering (k = 8) and the average was plotted.

The distinct duration of kinase activities at late phases after stimulation suggests regulation via transcriptionally induced feedback. To identify genes that potentially modulate kinase activity kinetics, we performed a comprehensive gene expression analysis via microarray using wild type, MEK inhibitor-pre-treated (WT + Inh), or IKKβ inactive (IKKβSA) cells following stimulation with BCR or CD40 for 0, 15, 30, 45, 60, or 90 min. We selected genes that showed significantly altered expression levels when compared to control cells (time 0). From this, 578 genes were selected (False Discovery Rate (FDR) < 0.05) (Fig. 2a and Supplementary Fig. S2). To explore the biological function of the selected genes, we employed a Gene Ontology (GO) enrichment analysis (Table 1). As expected, many genes that were significantly enriched exhibited functions known to contribute to immune- and signal-regulation. The most popular GO term was related to the positive and negative regulation of signals, which suggested possible roles of the selected genes in the regulation of signalling pathways. In our experimental setting, only three CD40-specific genes were identified (Fig. 2a), that is LOC768982, cation transport regulator homolog 1 (*CHAC1*), and TNF receptor-associated factor 5 (*TRAF5*), and that are not related to the regulation of IKK and ERK signalling^21^. In contrast, BCR-stimulation induced the up- and down-regulation of a large number of genes (511) over 90 min (Fig. 2a,b) and shared 13 genes in common with CD40 stimulation. Therefore, we performed k-means clustering on the BCR-regulated genes, which resulted in their subdivision into eight clusters based on their time-course profiles (Fig. 2c). Clusters 1 (26 genes), 2 (52 genes), 3 (93 genes), and 4 (56 genes) showed increasing gene expression with slightly different expression peak times. Notably, A20^28^, a typical feedback regulator of IKK and an early stimulus response gene in NF-κB signalling, was included in cluster 1, which showed an immediate increase after cell stimulation. Thus, we explored other potential feedback regulators by focusing on cluster 1 (the full list of genes is provided in Supplementary Dataset 1).

### Identification of feedback regulators

**Figure 3:**
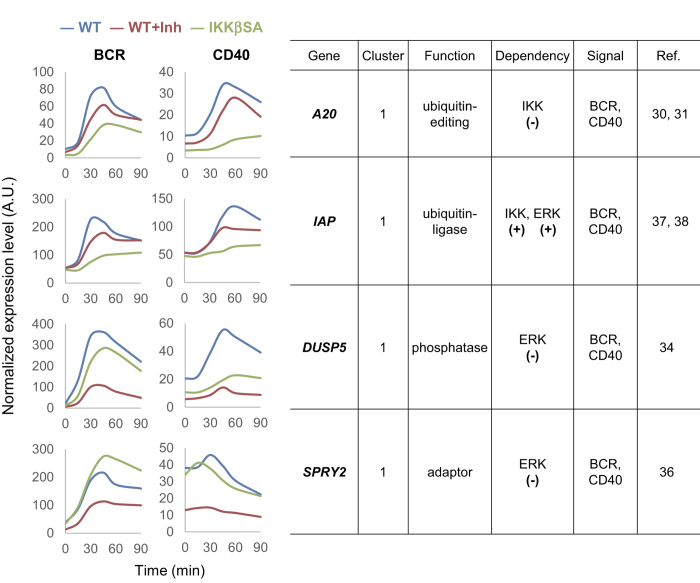
Gene expression dynamics of feedback regulator candidates. The normalized expression levels (arbitral unit; A.U.) from the data of BCR- (left columns; BCR) or CD40- (right columns; CD40) stimulated samples are plotted. WT, wild type cells (blue); WT + Inh, MEK inhibitor-treated wild type cells (red); IKKβSA, IKKβ inactive cells (green). Right table: Cluster, gene cluster number; Function, molecular function; Dependency, pathway dependency of gene expression with known regulatory roles: positive (+) or negative (−) for IKK or ERK activation; Signal, known gene function in BCR or CD40 signalling pathway; Ref, reference number.

Based on the literature and previously reported primary B cell gene expression data^29^, we identified four candidate genes in cluster 1 as potential feedback regulators for ERK or IKK signalling in BCR- and CD40-stimulated B cells (Fig. 3). In addition to A20, we identified two other well-known negative feedback regulators, sprouty homolog 2 (*SPRY2*) and *DUSP5*. A20 is a ubiquitin editing enzyme and is known to negatively control the activation dynamics of IKK-NF-κB signalling^30^,^31^. DUSP5 is a MAPK phosphatase that negatively regulates ERK activity and modulates both innate and adaptive immunity^32^,^33^,^34^. Sprouty proteins have been shown to inhibit ERK signalling^35^ and a member of the Sprouty family, mammalian SPRY2, has been suggested to regulate BCR-mediated ERK activity^36^. In addition to these protein genes, we identified an E3 ubiquitin ligase, IAP, as a novel feedback regulator. IAP has been suggested to positively regulate CD40-signalling-mediated IKK-NF-κB and ERK activation^37^,^38^. However, the role of IAP in BCR signalling remains unknown. Therefore, we examined the regulatory functions of IAP in BCR-signalling using IAP-deficient DT40 cells. Using this approach, our data clearly showed that IAP-deficient DT40 cells failed to sufficiently activate IKK and ERK following BCR engagement (Supplementary Fig. S3). Together, these findings suggest that IAP acts as a positive regulator in BCR-signalling.

### Mathematical modelling explains IAP feedback effects on the duration of IKK and ERK activity

**Figure 4:**
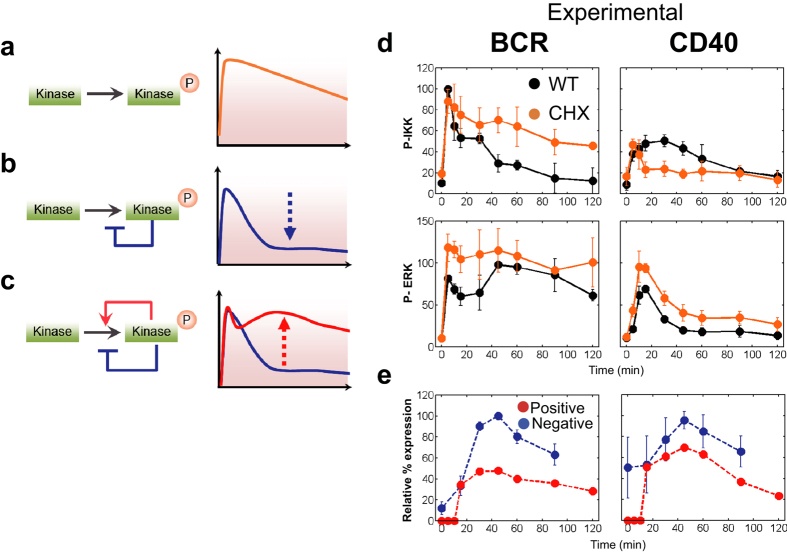
Assessment of transcriptional feedback regulation. (**a**–**c**) Scheme of feedback effects on kinase activity. Kinase activity without feedback (orange line) (**a**); kinase activity without positive feedback (blue line); hypothetical effect of negative feedback (blue dotted arrow) (**b**); kinase activity with positive and negative feedback (red line); hypothetical effect of positive feedback (red dotted arrow) (**c**). (**d**) The black dots represent the data of IKK or ERK activities in BCR or CD40-stimulated cells as shown in Fig. 1. The quantified activities of IKK and ERK in CHX pre-treated cells are represented as orange dots (+CHX). The activities were analysed by immuno blotting (Supplementary Fig. S3) as in Fig. 1. The data shown represent the means ± s.d. (n = 2). (**e**) Red circles depict the rate of newly synthesized IAP (Positive) relative protein amounts that were analysed by immuno blotting and calculated as described in Supplementary Fig. S4. The data shown represent the means ± s.d. (n = 2). The blue circle (Negative) indicates the averaged gene expression kinetics of *A20* for BCR-signalling and of *DUSP5* and *SPRY2* for CD40-signaling, respectively as displayed in Fig. 3. The values shown indicate values relative to the maximum for each gene expression. The dotted line represents the fitted curve as described in Methods.

**Figure 5:**
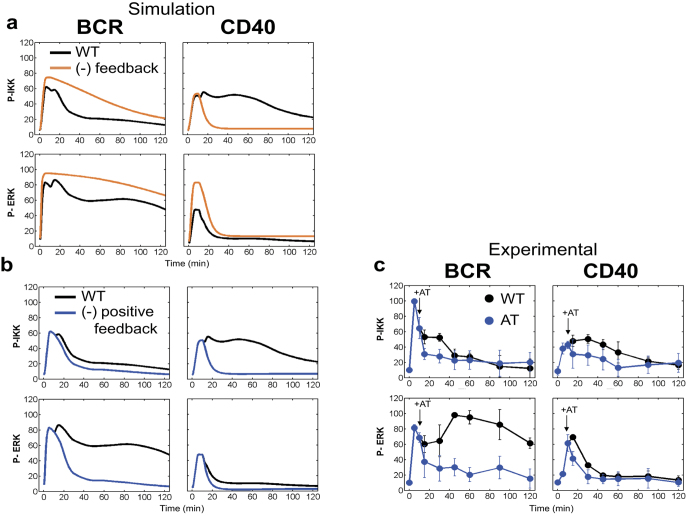
Simulation of IAP feedback effects on kinase activities. (**a**,**b**) Simulation data of model (A) are shown. IKK or ERK activities (black line); kinase activity without feedback ((−) feedback) (orange line) (**a**); kinase activity without positive feedback ((−) positive feedback) (blue line) (**b**). (**c**) The experimental data of IKK or ERK activities in BCR or CD40-stimulated cells are shown. The black dots represent cells without inhibitor (WT) as previously shown in Figs 1 and 4d. The blue dots indicate the quantified activities of IKK and ERK with IAP inhibitor (AT) added 10 min after stimulation (+AT, arrow). The activities were analysed by immuno blotting (Supplementary Fig. S3). The data shown represent the means ± s.d. (n = 2).

Genetic and biochemical approaches have limitations in identifying regulatory feedback loops, as genetic deletion in particular often ignores the development of biological networks over time^26^. In addition, as in the case under study, multiple positive (IAP) and negative (A20, DUSP, and SPRY2) feedback regulators combine to determine kinase activities. Therefore, we used a computational approach to evaluate the mechanisms underlying the IAP-mediated control of kinase activation dynamics. First, we constructed an ordinary differential equation (ODE)-based mathematical model (Figs 4 and 5, Supplementary Table S1; details of the model are described in the Methods section). Using this model (hereafter called model (A)), complied with Michaelis-Menten kinetics, we were able to describe the active states of kinases associated with three types of regulatory networks: those without feedback loops (Fig. 4a), those without a positive feedback loop (Fig. 4b), and those with positive and negative feedback loops (Fig. 4c). Kinetics parameters were obtained by fitting experimental time-course profiles of IKK and ERK activities in cycloheximide (CHX)-treated cells, IAP inhibitor-treated cells, and wild type cells (Figs 4d and 5c, and Supplementary Fig. S4), which corresponded to the three abovementioned regulatory networks, respectively (Fig. 4a–c). Since the effect of positive feedback is considered to be mainly dependent on IAP expression, we assumed that the reaction rate associated with this type of network would be proportional to the amount of newly synthesized IAP (Fig. 4e, red). The amount of synthesized IAP was obtained by subtracting the CHX-treated IAP protein value from the CHX-nontreated value (Supplementary Fig. S5). Further, we assumed that the negative feedback reaction rate was proportional to the averaged expression kinetics (Fig. 3) of the negative regulators (A20 for BCR-signalling and DUSP and SPRY2 for CD40-signalling) (Fig. 4e, blue). As shown in Fig. 5a, our simulated results fit well with the experimental data with/without transcriptional feedback (Fig. 4d). Furthermore, when we ran the simulation without the positive feedback loop, we observed activated kinase profiles that were similar to those observed experimentally in the presence of the IAP inhibitor (AT; AT-406) (Fig. 5b,c and Supplementary Fig. S4). Together, these results indicate that the dynamics of IKK activation in BCR-signalling is primarily shaped by negative feedback, while IKK activation dynamics in CD40-signalling is mainly determined by positive feedback. In contrast, our results on ERK activation dynamics implicate the involvement of two feedback loops in either BCR- or CD40-signalling. Thus, we demonstrated that the transcriptional feedback of IAP regulates kinase activity duration in B cells.

### IAP-mediated kinase activity duration is critical for late-phase gene expression

**Figure 6:**
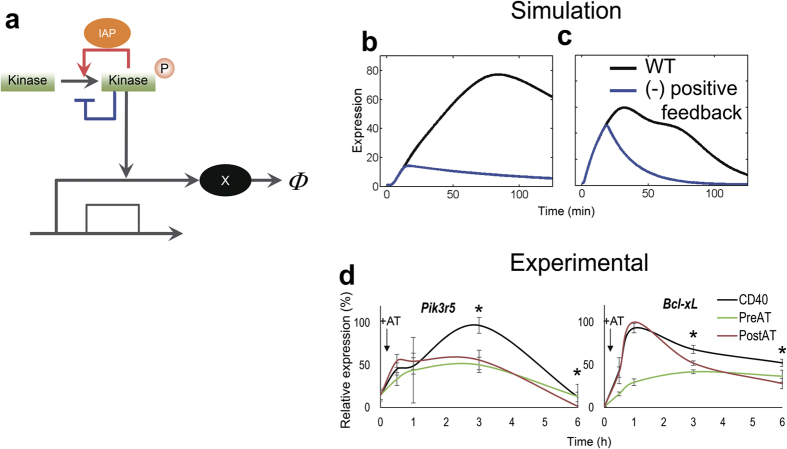
Evaluation of feedback effects on gene expression. (**a**) Schematic illustration of the simple transcriptional motif. (**b**) IKK activity-associated gene expression in CD40 stimulated cells. (**c**) ERK activity-associated gene expression in CD40 stimulated cells. The IKK activity and ERK activity of kinase in model (B) (**a**) were employed using the kinase activities of model (A) in Fig. 5b. (**d**) Quantitative analysis of gene expression examined by quantitative reverse transcriptional polymerase chain reaction. Cells were stimulated by anti-CD40 alone (CD40, black line), anti-CD40 pretreated with IAP inhibitor (PreAT, green line), and anti-CD40 plus IAP inhibitor added 10 min (+AT, allow) after stimulation (PostAT, red line). The data are represented as the means ± s.d. (n = 2). *P < 0.001, CD40 versus PostAT.

Quantitative information about signalling activity undergoes transcriptional regulation. Thus, to understand how IAP-regulated IKK and ERK activity durations affect downstream gene expression, we conducted a computational analysis using a simple transcriptional model^39^ (hereafter called model (B)) (Fig. 6a). Since we observed that IAP controlled IKK and ERK activation dynamics, signal inputs (*Kinase*) in model (B) were set as the IKK or ERK activities (Fig. 5b, right) used in model (A) with and without a positive feedback. Using this model, we observed that IKK input without positive feedback induced a low output (gene X expression) (Fig. 6b), whereas ERK signal without positive feedback showed transient gene expression (Fig. 6c). In comparison, input controlled by both positive and negative feedback led to a consistent level of amplitude and duration of gene expression (Fig. 6b,c, black line). We tested this prediction by examining the activation profiles of *Pik3r5* and *Bcl2l1* (*Bcl-x*_*L*_) in murine splenic B cells (Fig. 6d). To dissect any effects of positive feedback, we employed the following three conditions: CD40 stimulation alone, CD40 stimulation pre-treated with IAP inhibitor, and the addition of IAP inhibitor 10 min after CD40 stimulation. Expression of the two genes showed attenuated profiles over time in IAP inhibitor-pre-treated cells, although the difference between pre- and post-treated cells was not significant for *Pik3r5*. In comparison, IAP inhibition after CD40 treatment resulted in significantly lowered gene expression in the late phase of the examined time-course (Fig. 6d). Thus, our results indicated that the kinase activation dynamics that are controlled by IAP positive feedback are critical for late phase gene expression.

## Discussion

Using computational simulation and quantitative experiments, we revealed that IAP transcriptional feedback regulated the dynamics of ERK and IKK activity in B cells. In addition, we found that DUSP, A20, and SPRY2 might contribute to signalling and gene expression kinetics in B cells.

DUSPs are major players in the regulation of both BCR and CD40 signalling via negative feedback. Negative transcriptional regulation of ERK by DUSPs has been intensively characterized in previous studies^6^,^40^,^41^. In B cells, prolonged ERK activation has been reported to inhibit cell differentiation^34^; however, BCR signalling without cytokine or toll-like receptor co-stimulation, which induces ERK activation, cannot induce proper cell differentiation^42^,^43^,^44^,^45^. Consistent with this, ERK activation induced solely by BCR engagement showed sustained ERK activation in our system (Fig. 1), indicating that BCR-dependent DUSP5 induction might not be sufficient for the attenuation of ERK activity. Co-stimulatory cytokines (e.g., interleukin 2) might also be required to induce *DUSP* family gene expression that is sufficient enough to effect B cell differentiation^34^.

It is known that the NF-κB pathway is highly regulated via the attachment of K63-linked polyubiquitin chains, which lead to the recruitment of signalling molecules to induce signal transduction^46^. A20, CYLD, and A20-binding inhibitor of NF-κB (ABIN1) are ubiquitin-related enzymes or co-factors that terminate this signal through the disassembling of ubiquitin-anchored signal complexes^30^,^47^. In particular, the negative regulation of IKK’s activation dynamics appeared to be largely dependent on A20; both *CYLD* and *ABIN1* gene transcripts were expressed at much later time points (Supplementary Dataset 1). In fact, B cell-specific deletion of A20 in mice has been shown to sustain IKK activation in response to BCR and CD40 ligand binding, resulting in plasma cell hyperplasia and higher subsequent levels of serum immunoglobulins^32^,^48^,^49^. Together, these observations indicate that the signalling dynamics that are shaped by A20 are crucial for B cell biology.

**Figure 7:**
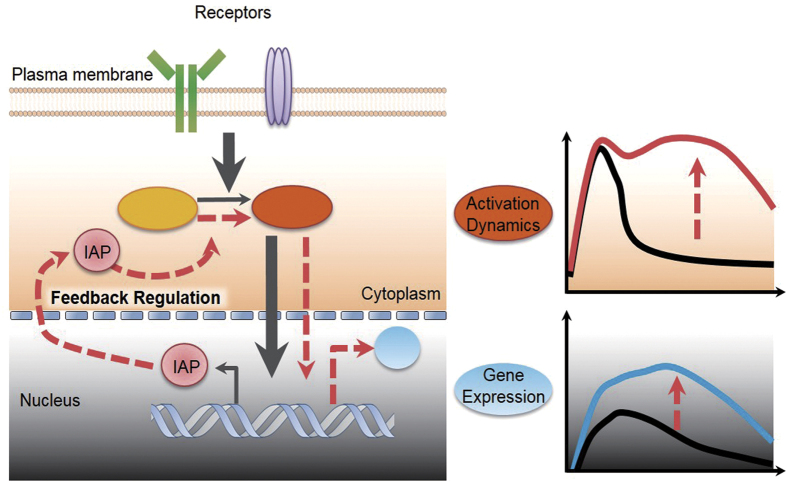
Schematic illustrating the transcriptional feedback of IAP. The signal (gray arrow) initiates the kinase activation (ellipse; yellow to orange). This induces early gene expression of IAP (red circle). IAP functions as a positive feedback regulator (orange dotted line) that effectively modulates the activation dynamics of the kinase (right upper box, orange line; intact activity of kinase, black line; activity without positive feedback, orange dotted arrow; predicted positive feedback effect). The duration of kinase activity mediated by IAP feedback generates certain gene (light blue circle) expression dynamics (right bottom box, gene expression trajectory with (light blue line) and without (black line) positive feedback effect).

Our mathematical model for kinase reactions supported the concept that IAP works as a positive regulator of IKK and ERK activation and that newly synthesized IAP prolongs their activities (Fig. 7). Furthermore, using experimentation and mathematical simulations, we demonstrated that IAP inhibition after CD40 stimulation lowered gene expression levels after 1 h. In the case of *Bcl-x*_*L*_, peak expression emerged after 1 h, and it is important to note that this kind of early gene expression might not be affected by newly expressed IAP. Accordingly, post-treatment with IAP inhibitor likely only affected *Pik3r5* expression (which was delayed) and later *Bcl-x*_*L*_ expression. Of note, we identified two patterns of effects of IAP on gene expression, one that involved elevated levels of expression (high amp-type) and one that involved sustained gene expression (sustained-type). Our simulation implied that the former was dependent on IKK input, while the latter relied on ERK input. In fact, *Pik3r5* was not induced in IKKβ-inactive cells (Supplementary Fig. S6). Moreover, up-regulation of *Bcl-x*_*L*_, which appeared to be dependent on ERK input, has been suggested to require ERK activation^50^. To confirm these findings, we tested whether forced expression of the dominant-positive forms of the IKKβ and MEK1, could restore the abovementioned patterns of mRNA expression. Indeed, high amp-type *Pik3r5* and sustained-type *Bcl2A1* expression, which were reduced in IAP-deficient DT40 cells, were rescued by the dominant-positive forms of IKKβ and MEK1, respectively (Supplementary Fig. S7).

The importance of IAP in B cell physiology has been highlighted by the finding that IAP-deficient B cells fail to mobilize B cell immunity^37^. Indeed, our current study revealed that IAP was required for ERK and IKK activation, which are involved in the regulation of BCR- and CD40-signalling via positive feedback; however, the site of IAP action remains unclear. IAP can interact with several proteins including ABINs, IKK, A20, NIK, and TRAFs^51^, and also mediates the canonical NF-κB pathway in TNF receptor signalling via K63 ubiquitination of receptor (TNFRSF)-interacting serine-threonine kinase 1 (RIP1) with the E2 ubiquitin ligase UBC5^46^,^51^,^52^. During CD40 signalling, IAPs induce K48 ubiquitination and degradation of the TRAF3 protein that initiates the release of TRAF2-MEKK1 and TRAF6-TAK1 signalling complexes into the cytoplasm. This process then leads to the activation of MAPK and NF-κB pathways. In the current study, CD40 signalling depicted a faster decrease in activation kinetics even though no transcriptional feedback effects were observed when compared to BCR signalling (Fig. 4d). Thus, we assumed that the ubiquitin-dependent degradation of the signal module progressed independently of gene expression, since IAP showed rapid decay in CHX-treated cells (Fig. 4d). Consistent with this assumption, proteasome inhibition has been reported to sustain activation of MAPK^38^.

Regarding BCR-signalling, the molecules which interact with IAP remain elusive. Here, we revealed that IAP-mediated inhibition of the IKK pathway had slightly different effects on BCR- and CD40-signalling, suggesting that IAP complex formation and its targets might differ (Fig. 5). Therefore, we predicted IAP-interacting proteins via network analysis of a public database (Supplementary Fig. S8). Since IAP is a K48 ubiquitin ligase, negative regulators of signalling, such as TRAF3 in CD40-signaling, could act as direct targets of the pathway^52^. In this context, A20, a negative regulator of IKK^28^ could be regarded as one potential target of BCR-signalling. Finally, although TRAF3 is associated with both IKK and ERK pathways in CD40-signalling, we failed to find a molecule which interacts with A20 in the ERK pathway. Thus, determining the link between IAP and the ERK cascade will require further study.

Since IAP positive feedback is critical for establishing gene expression profiles, information regarding feedback-specific IAP target molecules or modification sites will permit the control of gene expression dynamics. Furthermore, the ability to regulate gene expression is considered a promising approach in identifying pharmaceutical targets with minimal side effects^53^.

In conclusion, in this study we employed mathematical modelling to analyse the effects of transcriptional feedback – a key determinant of gene expression kinetics – on generating distinct dynamics during BCR- and CD40-mediated signalling.

## Materials and Methods

### Mice, cells, antibodies (Abs), and reagents

C57BL/6 mice from Charles River Laboratories International, Inc. were maintained under specific pathogen-free conditions and used at 8–12 wk of age. All protocols were approved by the RIKEN Animal Committee and all experiments were carried out in accordance with the approved guidelines. For western blots, splenic B cells were purified by depleting CD43^+^ cells with magnetic beads using AutoMACS (Miltenyi Biotec) and were cultured in Iscove’s modified Dulbecco’s medium supplemented with 10% foetal bovine serum and 1% penicillin-streptomycin.

*IKK*β knockout and knock-in (wild type and its S176/181A (SSAA) mutant; IKKβSA) cells have been described previously^54^. Wild type and mutant DT40 cells were cultured in Roswell Park Memorial Institute (RPMI) 1640 medium (Life Technologies) supplemented with 10% foetal calf serum, 1% chicken serum, 50 μM 2-mercaptoethanol (Wako), 4 mM L-glutamine, and antibiotics.

Abs specific for ERK, anti-phospho-ERK, anti-phospho-IκBα, and anti-phospho-IKKα/β were purchased from Cell Signaling Technology. The anti-chicken IgM mAb M4 was used for BCR stimulation^55^. Anti-mouse IgM mAb (α-μ) was obtained from Jackson Immuno Research, and anti-mouse CD40 from BD Biosciences. A GST-chicken-sCD154 fusion protein was used as the CD40 ligand. The mitogen activated kinase kinase (MEK) inhibitor, U0126 (Wako), was used at a concentration of 5 μM and the IAP inhibitor, AT, (AT-406; Active Biochem) was used at concentration of 1 μM.

IKK kinase assays were performed as described previously^55^. For protein detection, the ECL Plex fluorescent western blotting system and ImageQuant LAS 4000 (GE Healthcare) were used. Kinase activity was quantified from the intensities of the protein and phosphorylated protein bands using a Multi Gauge version2.2 (Fujifilm) densitometer as described previously^29^.

### Generation of IAP-deficient DT40 cells

A genomic clone of chicken *IAP* was obtained by polymerase chain reaction (PCR) using oligonucleotides designed by the NCBI database (Gene ID: 374012) as primers and chicken DT40 B cell genomic DNA as a template. The targeting constructs for chicken *IAP* were designed for the neomycin and histidinol drug-resistant gene cassettes to replace exons 1–8 of the chicken *IAP* gene, as shown in Supplementary Fig. 3a. These targeting vectors were sequentially transfected into DT40 cells, resulting in generation of chicken *IAP*-deficient DT40 cells as described elsewhere^54^,^55^.

### Microarray analysis

Cells were stimulated with 10 μg/ml anti-IgM (M4) or 6 μg/ml CD40 ligand for 15, 30, 45, 60, or 90 min using wild type DT40 cells. To identify ERK- or IKK-dependent transcripts, DT40 or IKKβSA cells, respectively, were pre-treated with MEK inhibitor 30 min prior to ligand stimulation. Untreated cells were used as the control. Total RNA was purified with the Qiagen RNeasy mini kit. GeneChip (Affymetrix GeneChip Chicken Genome Array) experiments were carried out according to the manufacturer’s protocol. Microarray data analysis was performed as described previously^29^. The microarray data were deposited at the NCBI Gene Expression Omnibus database (GEO ID: GSE59002).

### Gene expression analysis

Total RNA from murine splenic B cells purified by depleting CD43^+^ cells was collected by using the NucleoSpin RNA kit (MACHEREY-NAGEL GmbH & Co.) and subjected to cDNA synthesis and quantitative PCR using the KOD SYBR qPCR kit (TOYOBO Life Science) according to the manufacturer’s instructions. The primers used to detect the transcripts for mice were as follows: *Pik3r5* (5′-CAC TGT CAC TGT GCT GTT GCT GAA C-3′ and 5′-CCT CCA GCT CTT CTA TTG TCT TGG AC-3′); *Bcl2l1* (5′-TAG GAC TGA GGC CCC AGA AGA AAC TGA AGC-3′ and 5′-AGT TCA AAC TCA TCG CCT GCC TCT CTC AGC-3′); and glyceraldehyde-3-phosphate dehydrogenase (GAPDH, as a normalizing housekeeping gene) (5′-CAT GTT CCA GTA TGA CTC CAC TCA C-3′ and 5′-CTT CTC CAT GGT GGT GAA GAC ACC AGT AG-3′). For chicken, the primers were as follows: *Pik3r5* (5′- CAG AGG ACA GGA TCT ATC ATG CAC TG-3′ and 5′- CAT CAC CAG GTC GTA GTC ACA CTT CTC-3′); *Bcl2l1* (5′-TGA GCA GGT AGT GAA TGA ACT CTT CC-3′ and 5′-GCG TTG TTC CCA TAC AGA TCC ACA-3′); and *GAPDH* (5′- AGG TGC TGA GTA TGT TGT GGA GTC-3′ and 5′- GTG GTG CAC GAT GCA TTG CTG ACA AT-3′).

### Mathematical modeling

Computational simulations were performed as an ODE-based model (A) for the species (shown in the diagram of Fig. 4c), and comprised of the following equations:


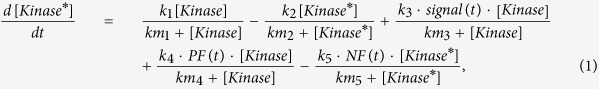



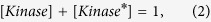


where *k*_*i*_ is a reaction rate constant, *km*_*i*_ is a Michaelis constant, *Kinase* is neutral IKK or ERK, Kinase* is active IKK or ERK, *signal*(*t*) is the time-course of upstream signalling activity, *PF*(*t*) is the time-course of the positive feedback regulator, IAP, and *NF*(*t*) is the averaged gene expression kinetics of the negative feedback regulators (A20 for BCR-signalling and DUSP and SPRY2 for CD40-signalling). Here, the expression of each gene is used relative to their own maximal values. The factor *signal(t)* was employed as a function according to our previous study^29^ as follows:


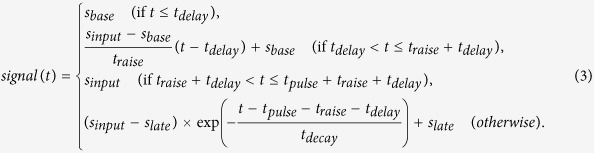


To reflect the gene expression kinetics and IAP synthesis data to the rate of feedback, time-courses of *NF*(t) and *PF(t)* were employed as interpolations of the relative expression profiles of the negative regulators (Fig. 4e) and newly synthesized IAP protein expression data (Fig. 4e and Supplementary Fig. S4), respectively, using the method “pchip” of the function *interp1* in MATLAB R2014a (MathWorks, Natick MA). The simulations for Fig. 4a,b changed the parameters *k*_*4*_ and *k*_*5*_ to zeros and the parameter *k*_*4*_ to zero, respectively.

Model (B) of the simple motif for gene expression, as shown in the diagram of Fig. 6a, comprised the following equation:


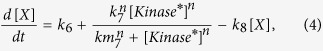


where *X* is a gene and *n* is the Hill coefficient. Simulations were performed using the function *ode15s* of MATLAB R2014a. All parameters can be found in Supplementary Tables S1 and S2.

## Additional Information

**How to cite this article**: Shinohara, H. *et al.* Stimulus-Dependent Inhibitor of Apoptosis Protein Expression Prolongs the Duration of B Cell Signalling. *Sci. Rep.* **6**, 27706; doi: 10.1038/srep27706 (2016).

## Supplementary Material

Supplementary Information

Supplementary Dataset 1

Supplementary Dataset 2

## Figures and Tables

**Table 1:** Gene enrichment analysis.

Gene Ontology					
		BCR stimulation		CD40 stimulation	
Name	ID	FDR	Gene symbol	FDR	Gene symbol
Biological regulation	GO:0065007	0.006	*ASB9*, *BCL2A1*, *BIRC2*, *BLNK*, *BTG1*, *CLK2*, *DUSP4*, *EGR1*, *EIF2C3*, *ELL*, *GNAI1*, *HHEX*, *HMOX1*, *MARCKSL1*, *MITF*, *MYD88*, *NFKB1*, *NFKBIA*, *PIK3AP1*, *PPARG*, *PSEN1*, *RAB9A*, *RASGEF1A*, *REL*, *RGS2*, *SIK1*, *SOCS3*, *SPRY2*, *USP47*	0.0148	*NFKB1, NFKBIA, RASGEF1A, RGS9BP, TRAF5*
Cellular process	GO:0009987	0.021	*AMD1*, *ASB9*, *ATP1B1*, *B3GNT2*, *BIRC2*, *BLNK*, *CARS*, *CLK2*, *DUSP4*, *EGR1*, *EIF2C3*, *ELL*, *GNAI1*, *HHEX*, *HMGCS1*, *HMOX1*, *LOC395787*, *MITF*, *MYD88*, *NCDN*, *NFKB1*, *NFKBIA*, *ODC1*, *PGS1*, *PIK3AP1*, *PPARG*, *PSEN1*, *RAB9A*, *RASGEF1A*, *RBM38*, *REL*, *RGS2*, *SIK1*, *SOCS3*, *USP47*	0.1371	*B3GNT2, NFKB1, NFKBIA, RASGEF1A*
Immune system process	GO:0002376	0.007	*BLNK*, *EGR1*, *IL8*, *MITF*, *MYD88*, *NFKB1*, *NFKBIA*, *PPARG*, *PSEN1*	0.0259	*NFKB1, NFKBIA*

The representative results of Gene Ontology (GO) analysis providing the terms of biological processes. The complete list of GO terms is presented in Supplementary Dataset 2. FDR, false discovery rate. *BIRC2*, also known as *cIAP1*, is called *IAP* in the text.
