# Supplementary material for: Stimulus-Dependent Inhibitor of Apoptosis Protein Expression Prolongs the Duration of B Cell Signalling
Source: Sci Rep. 2016 Jun 9;6:27706. doi: 10.1038/srep27706 (PMC4899755; doi:10.1038/srep27706)
Supplement: Supplementary Information [file srep27706-s1.pdf]

Supplementary Material

Stimulus-Dependent Inhibitor of Apoptosis Protein Expression Prolongs the Duration of B Cell Signalling

Hisaaki Shinohara*, Kentaro Inoue, Noriko Yumoto, Takeshi Nagashima & Mariko Okada-Hatakeyama*

Laboratory for Integrated Cellular Systems, RIKEN Center for Integrative Medical Sciences (IMS), Yokohama, Kanagawa 230-0045, Japan.

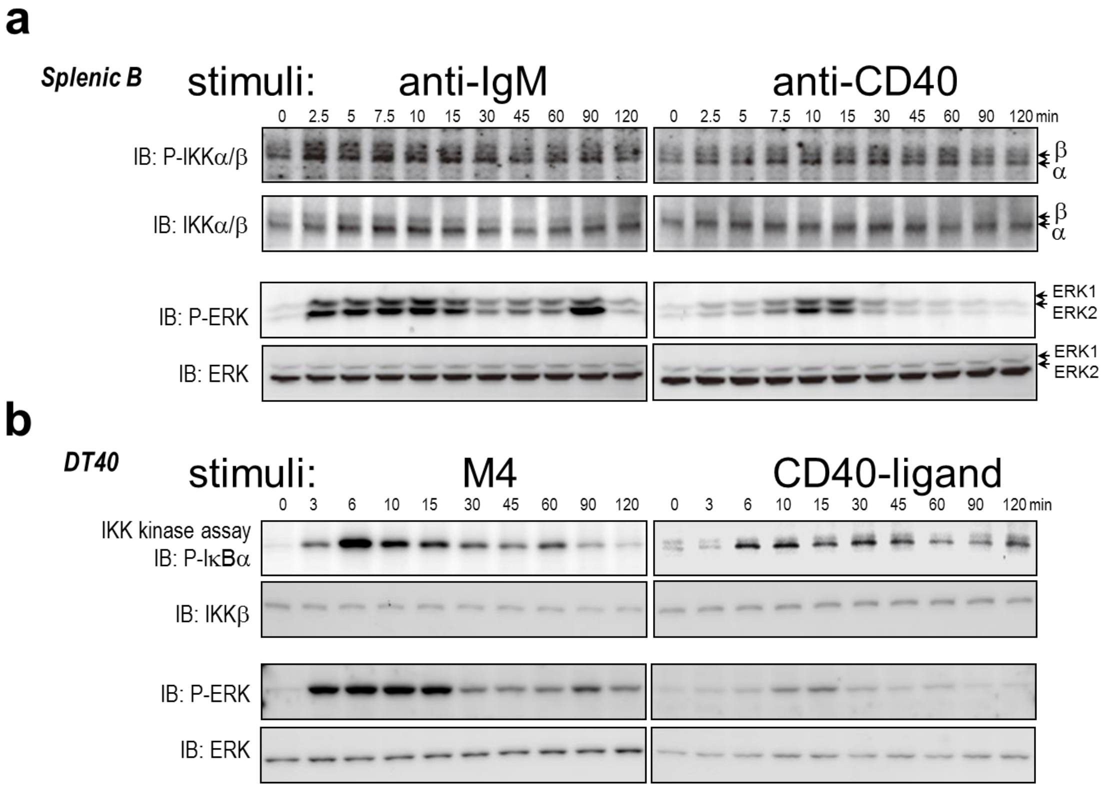

Supplementary Figure S1. Activation dynamics assessed by immuno blotting. (**a**) Splenic B cells (Splenic B) from mice were simulated with 10 μg/ml anti-mouse IgM or 4 μg/ml anti-mouse CD40 over the indicated time period. Whole cell lysates were analysed by immuno blotting with anti-phospho-IKK or anti-phospho-ERK polyclonal Abs (n = 2). Quantified data are displayed in Fig. 1. (**b**) DT40 wild type cells (DT40) were simulated with 10 μg/ml anti-chicken IgM (M4) or 6 μg/ml CD40 ligand over the indicated time period. For IKK and ERK activity, IKK kinase assays were performed and its activity was measured by the phosphorylation of GST-IκBα as a substrate and detected by anti-phospho-IκBα mAb; ERK activities were determined using the same procedures as in (**a**) (n = 2).

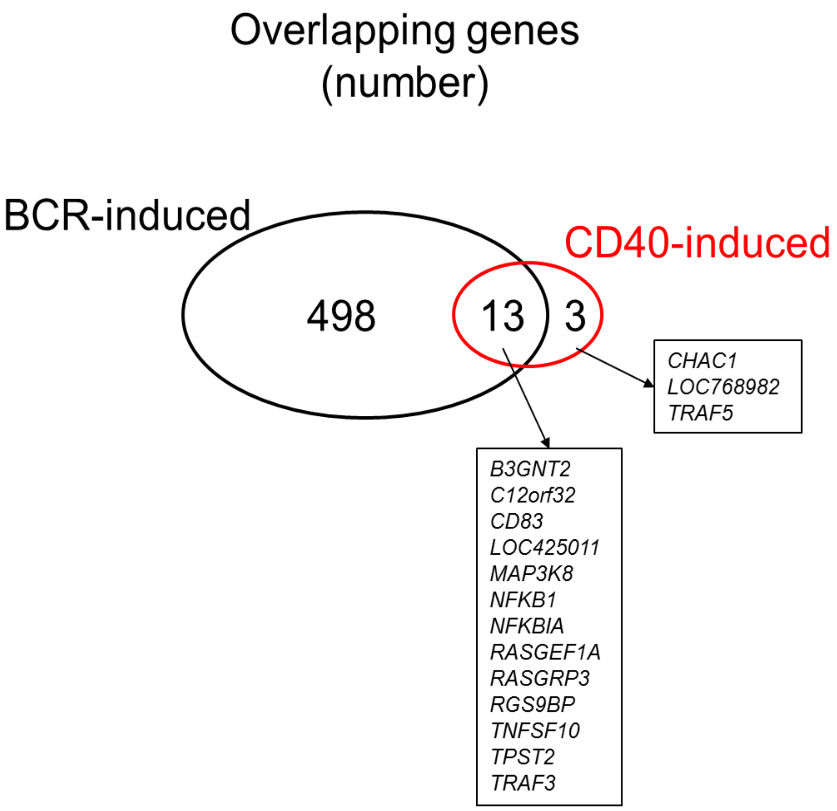

Supplementary Figure S2. Overlapping genes between BCR- and CD40-induced. DT40 B cells were stimulated with anti-IgM or CD40L and subjected to microarray expression analysis related to Figure 2A. Venn diagram shows numbers of genes expressed by anti-IgM treated (BCR-induced) and CD40L ligation (CD40-induced).

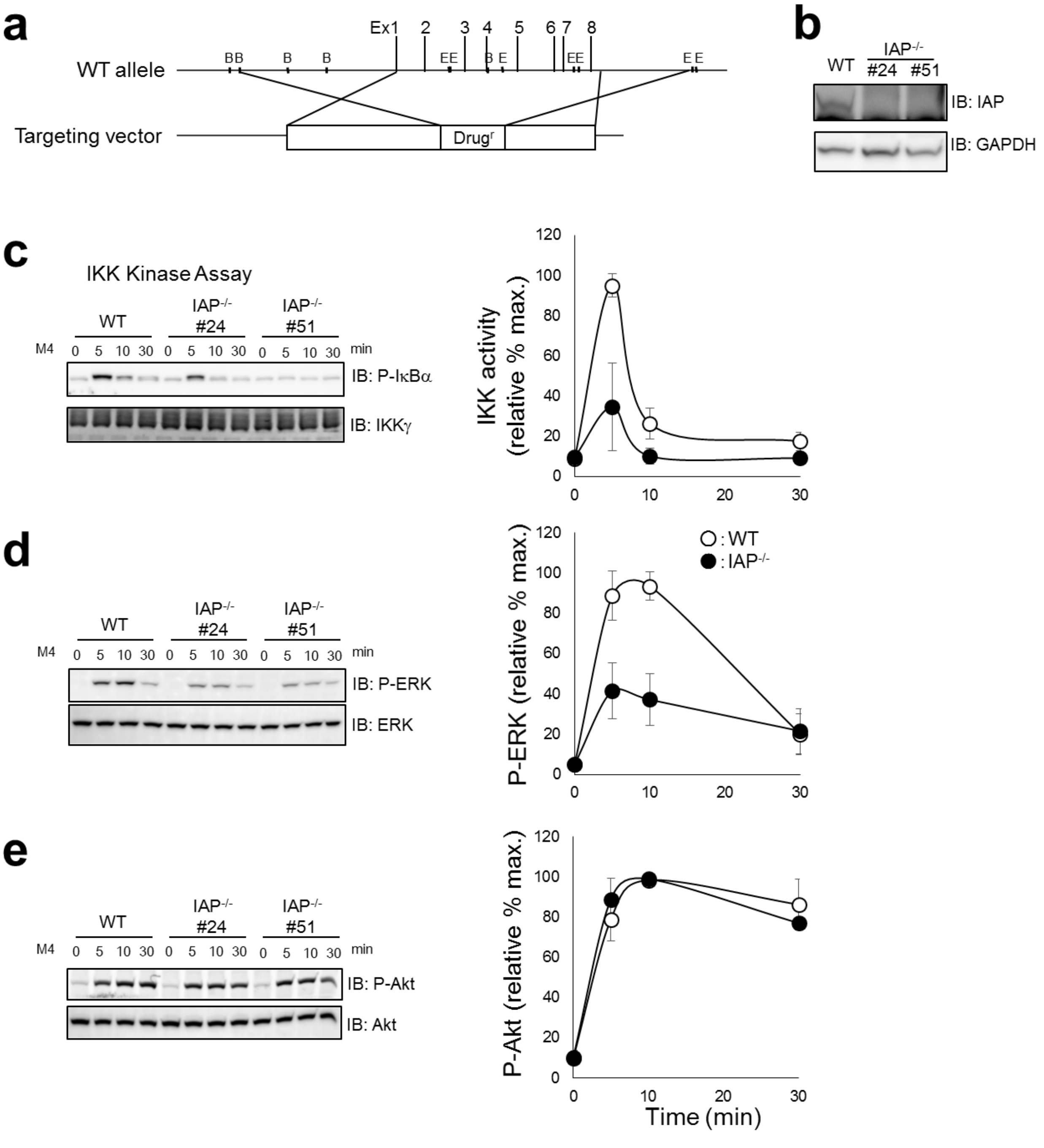

Supplementary Figure S3. IAP is a positive regulator for BCR-induced IKK and ERK activation. BCR-mediated IKK and ERK activation in DT40 wild type (WT) and chicken-IAP-deficient DT40 B cells (IAP$^{-/-}$). (**a**) The targeting strategy of chicken IAP. Drug^{r} indicates drug resistant gene cassette. E; EcoRI, B; BamHI. (**b**) IAP protein expression checked by immunoblotting. Two clone of IAP$^{-/-}$ were analyzed (#24, #51).

GAPDH blot represents loading control. (c-e) The following experiments were performed three independent (n=3), and representative data are shown. (c) For IKK activation, cells were subjected to an IKK kinase assay detected by phosphorylation of GST-IκBα. IKKγ was blotted as a loading control. The graph depicted quantitated IKK activity as previously described in Figure 1. (**d**) The phosphorylation of ERK was analysed by immuno blotting with an anti-phospho-ERK Ab. The ERK blot is a loading control. The graph represented the quantitated phosphorylated ERK (P-ERK). (**e**) The phosphorylation of Akt was analysed by immuno blotting with an anti-phospho-Akt (serine 473) Ab. The Akt is used as a loading control. The graph represented the quantitated phosphorylated Akt (P-Akt).

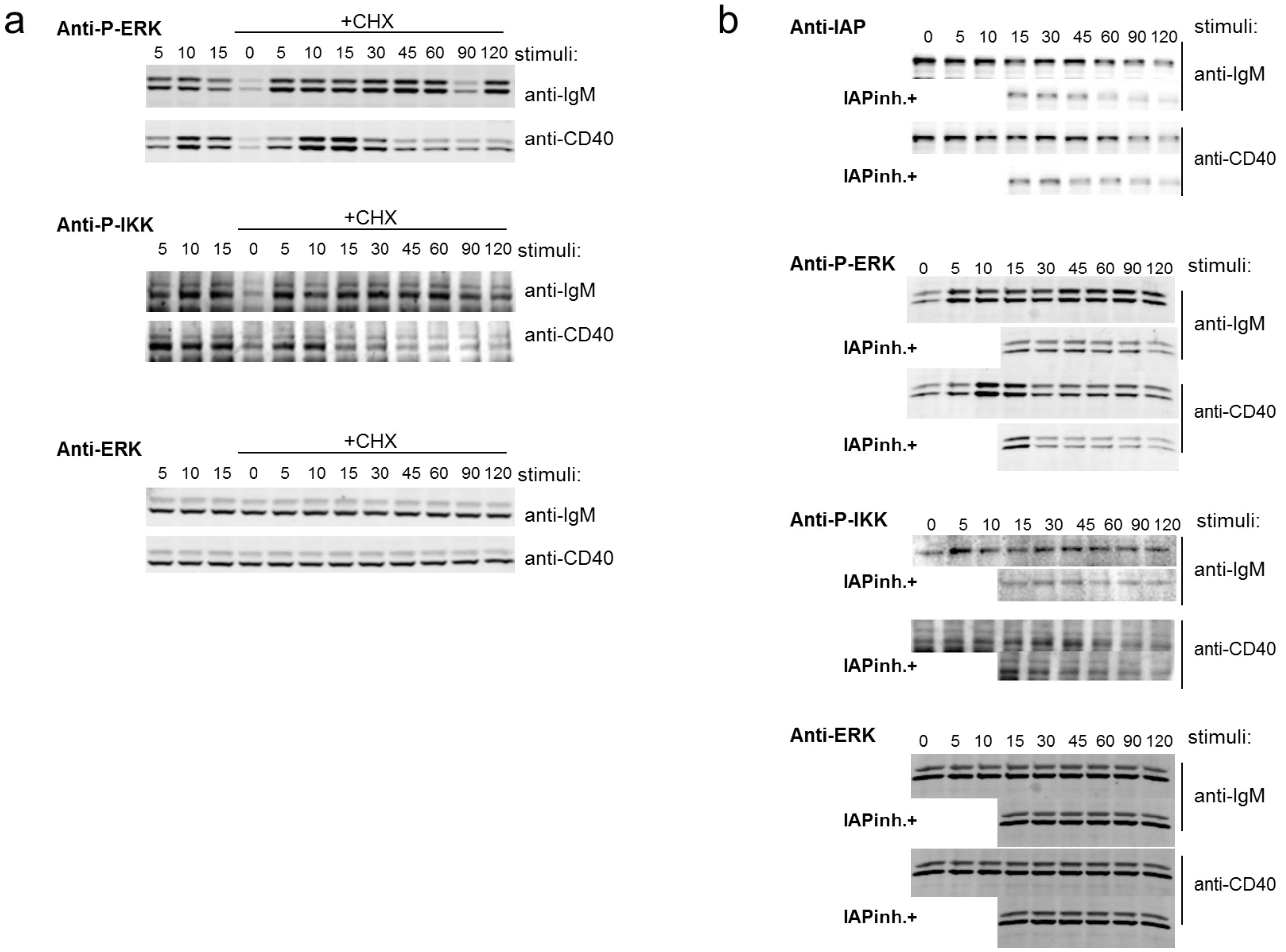

Supplementary Figure S4. Feedback effects assessed by immuno blotting. Whole cell lysates were analysed by immuno blotting with anti-phospho-IKK or anti-phospho-ERK polyclonal Abs as previously described in Supplementary Fig. S1. Representative data from at least two independent experiments are shown. The ERK signal is used as a loading control. The quantified data are displayed in Fig. 4. (**a**) Splenic B cells were simulated with 10 μg/ml anti-mouse IgM or 4 μg/ml anti-mouse CD40 for the indicated time period. +CHX indicates cycloheximide pretreated at a concentration of 10 μM. The quantified data are shown in Fig. 4d. (**b**) IAP inhibitor (AT-406) was used at a concentration of 100 μM (IAPinh+) and added 10 min after stimulation with 10 μg/ml anti-mouse IgM or 4 μg/ml anti-mouse CD40. The quantified data are shown in Fig. 5c.

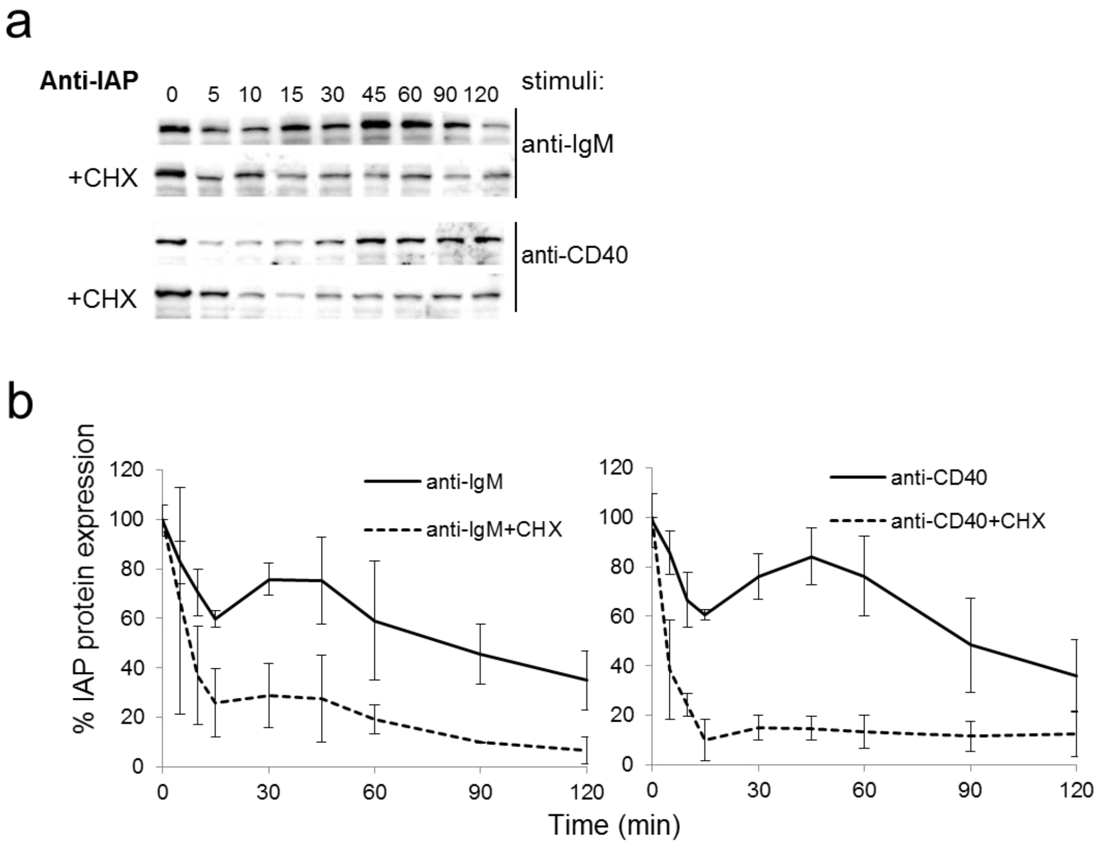

Supplementary Figure S5. The estimation of newly synthesized IAP protein. (**a**) Whole cell lysates were analysed by immuno blotting with an anti-IAP polyclonal Ab. Splenic B cells were simulated with 10 μg/ml anti-mouse IgM or 4 μg/ml anti-mouse CD40 over the indicated time period. +CHX indicates cycloheximide pretreated at a concentration of 10 μM. Representative data from at least two independent experiments are shown. (**b**) Relative amounts of IAP protein in each sample were calculated as % values for the control cells without stimulus (time point 0) for the cells with (dotted line) or without (black line) CHX. The newly synthesized IAP (Fig. 4e, positive) was calculated as: [synthesized IAP] = [anti-IgM/anti-CD40] – [anti-IgM/anti-CD40+CHX].

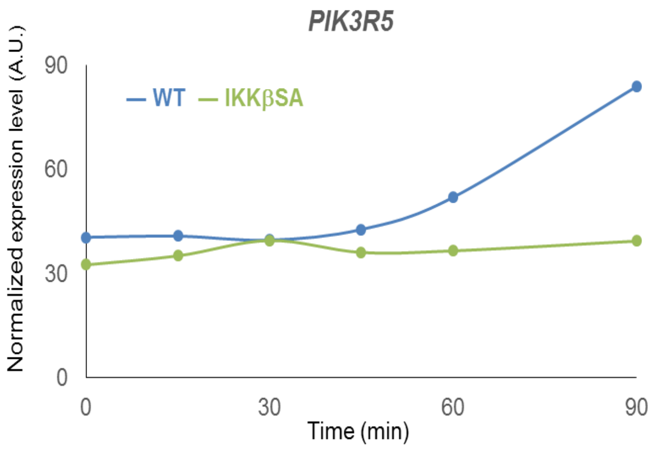

Supplementary Figure S6. CD40 induced *Pik3r5* gene expression in IKKβ inactive cells. The normalized expression levels (arbitral unit; A.U.) of *Pik3r5* were obtained from microarray data of the CD40-stimulated cells shown in Fig.2 and plotted on the gragh. WT, wild type cells (blue); IKKβSA, IKKβ inactive cells (green).

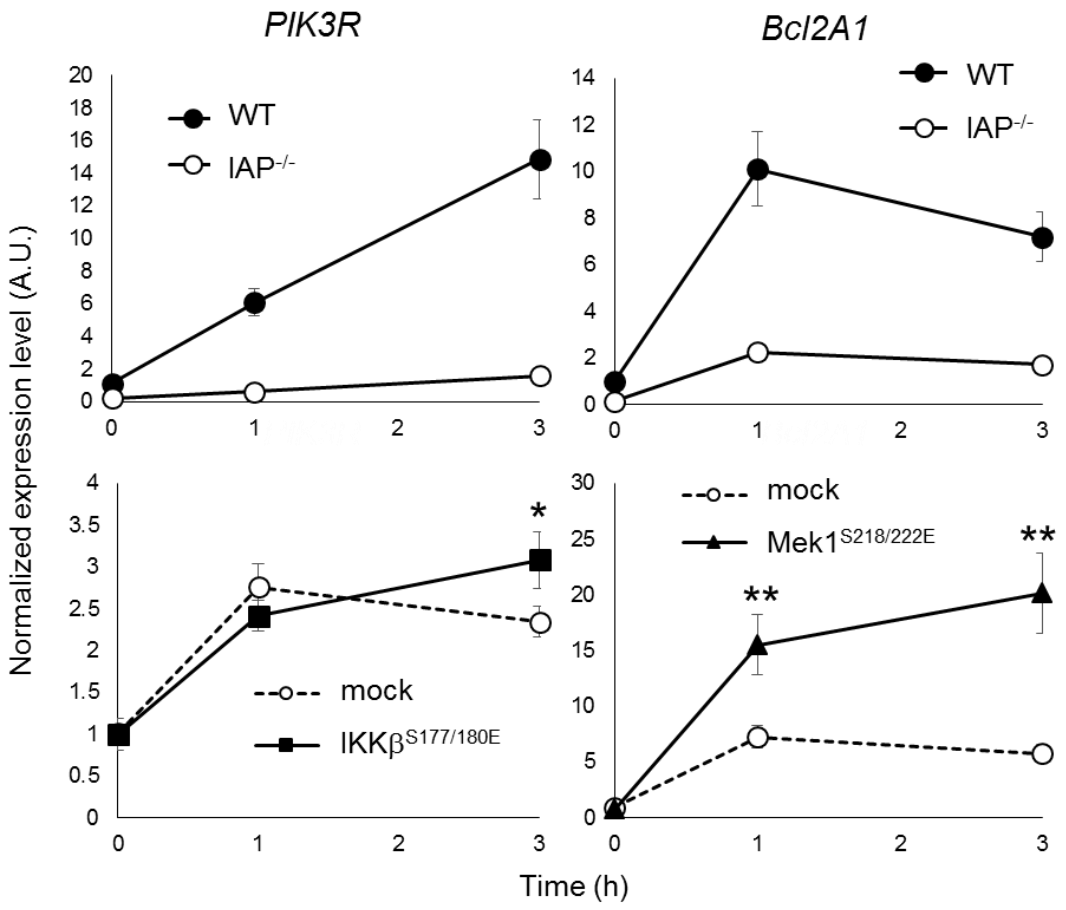

Supplementary Figure S7. The dominant positive form of IKKβ and MEK1 rescue the mRNA expression. The constitutive activation form of IKKβ (IKKβ$^{S177/180E}$) or ERK kinase, Mek1 (Mek1$^{S218/222E}$) were forced to express in IAP$^{-/-}$ DT40 cells. Cells were stimulated anti-IgM (M4, 10 μg/ml) at indicated time. The mRNA expression levels (arbitral unit; A.U.) were assessed by quantitative RT-PCR and normalized with GAPDH. WT, wild type cells (black circle); IAP$^{-/-}$, IAP-deficient cells (white circle); mock, empty vector control in IAP$^{-/-}$ (white circle with dotted line); IKKβ$^{S177/180E}$, dominant positive IKKβ expressed in IAP$^{-/-}$ (square); Mek1$^{S218/222E}$, dominant positive Mek1 expressed in IAP$^{-/-}$ (triangle). The data are represented as the means ± s.d. (n = 2). *$P < 0.03$, **$P < 0.01$, versus mock.

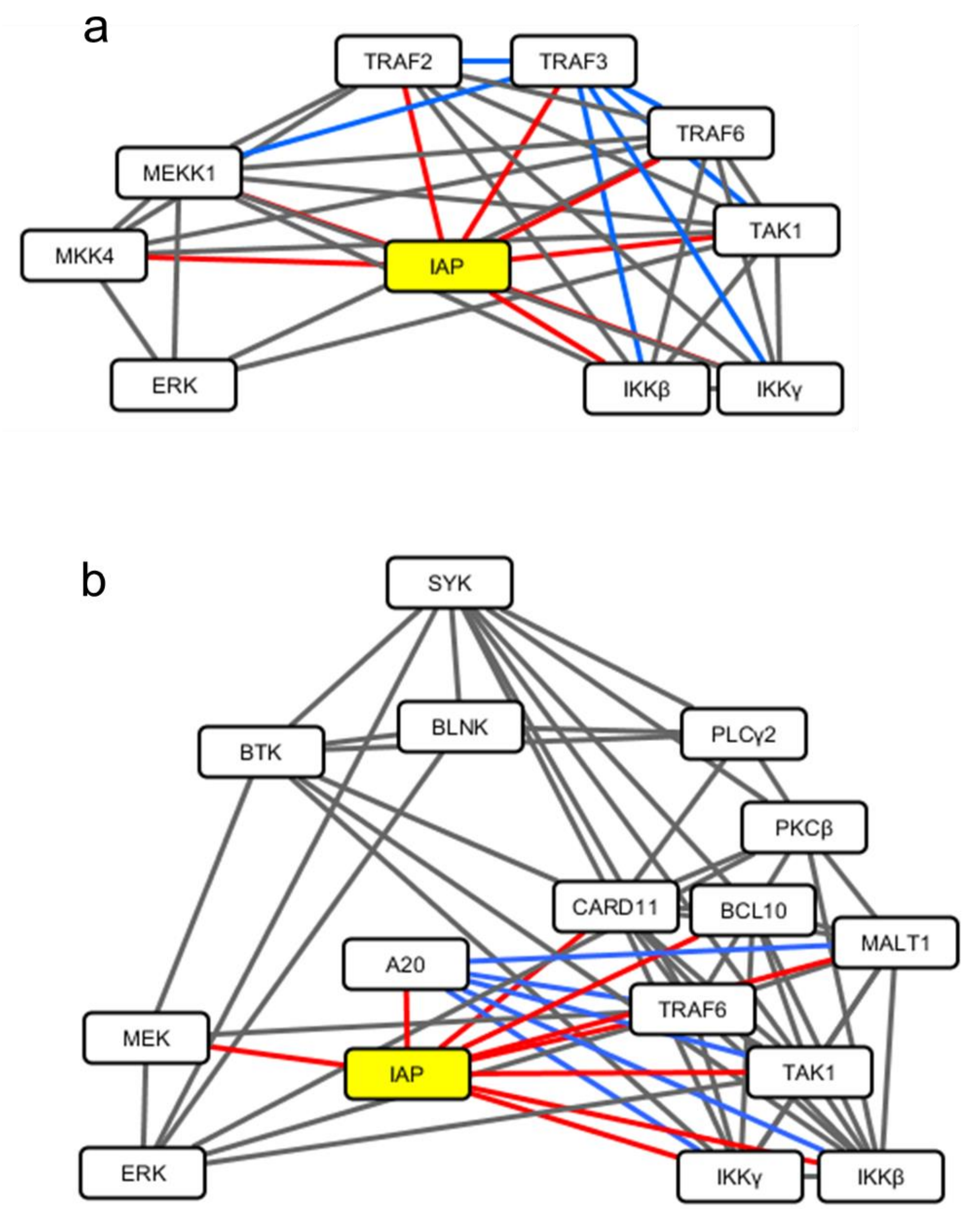

Supplementary Figure S8. Network analysis of IAP signaling pathway. IAP and its interacting proteins in the CD40 (a) and BCR (b) signaling were shown. The lines indicate protein-protein interactions. The red lines indicate interactions between IAP and its interacting proteins. The blue lines are TRAF3 interaction in CD40 signaling (a), and A20 interaction in BCR signaling (b), respectively. Signaling molecules in BCR and CD40 pathways are manually curated and protein interaction information was extracted from the STRING[1] database and drawn by Cytoscape[2].

Supplementary Table S1. Parameters of model (A) for each kinase activation.

Parameter name	BCR		CD40	
	IKK	ERK	IKK	ERK
k_1	0.1	0.1	0.1	0.1
k_2	1	1	1	1
k_3	2	10	1	3
k_4	1	20	1	1
k_5	2	10	1	3
km_1	1	1	1	1
km_2	1	1	1	1
km_3	1	1	1	1
km_4	0.5	0.5	0.5	0.5
km_5	0.5	0.5	0.5	0.5
sinput	1		1	
sbase	0.01		0.01	
tplulse	5		5	
traise	5		5	
tdecay	50		5	
tdelay	0		0	
slate	0.05		0.05	

Supplementary Table S2. Parameters of model (B).

Parameter name	CD40-IKK	CD40-ERK
k_6	0.01	0.01
k_7	2	2
k_8	0.01	0.05
km_7	0.4	0.1
n	5	10

Supplementary references.

1 Szklarczyk, D. *et al.* STRING v10: protein-protein interaction networks, integrated over the tree of life. *Nucleic Acids Res* **43**, D447-452, doi:10.1093/nar/gku1003 (2015).

2 Shannon, P. *et al.* Cytoscape: a software environment for integrated models of biomolecular interaction networks. *Genome Res* **13**, 2498-2504, doi:10.1101/gr.1239303 (2003).
